# Draft Genome Sequence of *Saccharomycopsis fodiens* CBS 8332, a Necrotrophic Mycoparasite with Biocontrol Potential

**DOI:** 10.1128/genomeA.01278-17

**Published:** 2017-11-16

**Authors:** Klara Junker, Ana Hesselbart, Jürgen Wendland

**Affiliations:** a Carlsberg Research Laboratory, Yeast & Fermentation, Copenhagen V, Denmark; b Department of Bioengineering Sciences, Research Group of Microbiology, Functional Yeast Genomics, Vrije Universiteit Brussel, Brussels, Belgium

## Abstract

*Saccharomycopsis fodiens* is an ascomycetous necrotrophic mycoparasite. Predator-prey interaction leads to killing of the host cell by a penetration peg and utilization of cell content by the predator. Here, we report the 14.9-Mb *S. fodiens* draft genome sequence assembled into 9 large scaffolds and 13 minor scaffolds (<20 kb).

## GENOME ANNOUNCEMENT

*Saccharomycopsis fodiens* is a member of the sole genus in the family *Saccharomycopsidaceae* (1). Previously, this genus gained attention due to the ability of *Saccharomycopsis fibuligera* to degrade starch by producing a suite of enzymes, including α-amylase, glucoamylase, β-glucosidase, and acid protease (2). Subsequently, *S. fibuligera* genes encoding these enzymes were used in heterologous hosts, such as *Saccharomyces cerevisiae* and *Yarrowia lipolytica* (3–7). This interest in using *S. fibuligera* also spurred genome sequencing of this yeast (8).

A totally different aspect of *Saccharomycopsis* yeast biology is the ability to act as necrotrophic mycoparasites, killing other fungi via penetration pegs (9). The broad host range includes both ascomycetes and basidiomycetes, yeasts, and filamentous fungi (10). This broad host range apparently enables *Saccharomycopsis* predators to use their penetration pegs like Swiss army knives. Due to their potential use as biocontrol agents, we are interested in elucidating the molecular biology of their predation. Here, we report the draft genome sequence of *S. fodiens*. This strain was isolated in 1995 in Queensland, Australia, and was the first saccharomycete for which predacious behavior was described (11).

With this draft genome sequence, we can now enter comparative genome biology of *Saccharomycopsis* species and provide genomic insight for strain improvements of fermentation traits. This knowledge will also fuel our understanding of the predatory behaviors of different *Saccharomycopsis* species.

The draft genome sequence of *S. fodiens* (CBS 8332 = NRRL Y-48786 = UWOPS 95-697.4) we report here was determined using Illumina MiSeq paired-end read sequencing. The *S. fodiens* strain was grown overnight at 30°C in rich medium (YPD, 1% yeast extract, 2% casein peptone, and 2% dextrose). DNA extraction and sequencing were carried out by LGC Genomics (Berlin, Germany). Two paired-end libraries were sequenced, producing 7,771,134 raw reads. These were quality processed and trimmed, resulting in 7,687,844 high-quality reads. The 250-bp paired-end library with short fragments produced 4,605,178 quality reads, and a further 3,082,666 high-quality reads were obtained from an 8-kb mate pair library. All quality-controlled reads were assembled using Bowtie 2 version 2.1.0. Initially, 90 contigs were assembled, with a total of 14,879,925 bp and an *N*_50_ of 508,788 bp. This assembly was refined by scaffolding these contigs into 9 scaffolds (>20 kb) comprising 14,908,178 bp and an *N*_50_ of 2,606,857 bp. The average GC content in these scaffolds is 51.5%, which is remarkably high compared with that of other ascomycetous yeasts. Additionally, 13 scaffolds with fewer than 12 kb were generated. The longest scaffold is 2,724,259 in size.

The large scaffolds were compared with those of the *S. cerevisiae* genome using BLASTX. This identified 4,725 hits (E value < 1e-10). BLASTX searches against the nonredundant database at NCBI (https://blast.ncbi.nlm.nih.gov/Blast.cgi) generated a further 373 hits. As was observed for *S. fibuligera*, also *S. fodiens* lacks genes required for sulfate uptake and assimilation (8). Running tRNAscan on the scaffolds identified 162 tRNA genes (12).

### Accession number(s).

This whole-genome shotgun project has been deposited in DDBJ/ENA/GenBank under the accession no. JNFV00000000. The version described in this paper is the first version, JNFV01000000.

## References

[1] Suh SO, Blackwell M, Kurtzman CP, Lachance MA 2006 Phylogenetics of *Saccharomycetales*, the ascomycete yeasts. Mycologia 98:1006–1017. 17486976 10.3852/mycologia.98.6.1006

[2] Chi Z, Chi Z, Liu G, Wang F, Ju L, Zhang T 2009 *Saccharomycopsis fibuligera* and its applications in biotechnology. Biotechnol Adv 27:423–431. doi:10.1016/j.biotechadv.2009.03.003. 19328842

[3] Gurgu L, Lafraya Á, Polaina J, Marín-Navarro J 2011 Fermentation of cellobiose to ethanol by industrial *Saccharomyces* strains carrying the beta-glucosidase gene (BGL1) from *Saccharomycopsis fibuligera*. Bioresour Technol 102:5229–5236. doi:10.1016/j.biortech.2011.01.062. 21324680

[4] Tang H, Hou J, Shen Y, Xu L, Yang H, Fang X, Bao X 2013 High beta-glucosidase secretion in *Saccharomyces cerevisiae* improves the efficiency of cellulase hydrolysis and ethanol production in simultaneous saccharification and fermentation. J Microbiol Biotechnol 23:1577–1585. doi:10.4014/jmb.1305.05011. 23928840

[5] Yu XJ, Chi Z, Wang F, Li J, Chi ZM, Madzak C 2013 Expression of the acid protease gene from *Saccharomycopsis fibuligera* in the marine-derived *Yarrowia lipolytica* for both milk clotting and single cell protein production. Appl Biochem Biotechnol 169:1993–2003. doi:10.1007/s12010-013-0118-1. 23354502

[6] Casa-Villegas M, Marín-Navarro J, Polaina J 2017 Synergies in coupled hydrolysis and fermentation of cellulose using a *Trichoderma reesei* enzyme preparation and a recombinant *Saccharomyces cerevisiae* strain. World J Microbiol Biotechnol 33:140. doi:10.1007/s11274-017-2308-4. 28589508

[7] Lee CR, Sung BH, Lim KM, Kim MJ, Sohn MJ, Bae JH, Sohn JH 2017 Co-fermentation using recombinant *Saccharomyces cerevisiae* yeast strains hyper-secreting different cellulases for the production of cellulosic bioethanol. Sci Rep 7:4428. doi:10.1038/s41598-017-04815-1. 28667330 PMC5493647

[8] Choo JH, Hong CP, Lim JY, Seo JA, Kim YS, Lee DW, Park SG, Lee GW, Carroll E, Lee YW, Kang HA 2016 Whole-genome *de novo* sequencing, combined with RNA-Seq analysis, reveals unique genome and physiological features of the amylolytic yeast *Saccharomycopsis fibuligera* and its interspecies hybrid. Biotechnol Biofuels 9:246. doi:10.1186/s13068-016-0653-4. 27872659 PMC5106798

[9] Lachance MA, Pang WM 1997 Predacious yeasts. Yeast 13:225–232. doi:10.1002/(SICI)1097-0061(19970315)13:3<225::AID-YEA87>3.0.CO;2-I. 9090051

[10] Lachance MA, Pupovac-Velikonja A, Natarajan S, Schlag-Edler B 2000 Nutrition and phylogeny of predacious yeasts. Can J Microbiol 46:495–505. doi:10.1139/w00-021. 10913970

[11] Lachance MA, Rosa CA, Carvajal EJ, Freitas LF, Bowles JM 2012 *Saccharomycopsis fodiens* sp. nov., a rare predacious yeast from three distant localities. Int J Syst Evol Microbiol 62:2793–2798. doi:10.1099/ijs.0.043109-0. 22691435

[12] Lowe TM, Eddy SR 1997 tRNAscan-SE: a program for improved detection of transfer RNA genes in genomic sequence. Nucleic Acids Res 25:955–964. 9023104 10.1093/nar/25.5.955 PMC146525

